# Evolutionary History of Lake Tanganyika's Predatory Deepwater Cichlids

**DOI:** 10.1155/2012/716209

**Published:** 2012-05-17

**Authors:** Paul C. Kirchberger, Kristina M. Sefc, Christian Sturmbauer, Stephan Koblmüller

**Affiliations:** Department of Zoology, Karl-Franzens-University Graz, Universitätsplatz 2, 8010 Graz, Austria

## Abstract

Hybridization among littoral cichlid species in Lake Tanganyika was inferred in several molecular phylogenetic studies. The phenomenon is generally attributed to the lake level-induced shoreline and habitat changes. These allow for allopatric divergence of geographically fragmented populations alternating with locally restricted secondary contact and introgression between incompletely isolated taxa. In contrast, the deepwater habitat is characterized by weak geographic structure and a high potential for gene flow, which may explain the lower species richness of deepwater than littoral lineages. For the same reason, divergent deepwater lineages should have evolved strong intrinsic reproductive isolation already in the incipient stages of diversification, and, consequently, hybridization among established lineages should have been less frequent than in littoral lineages. We test this hypothesis in the endemic Lake Tanganyika deepwater cichlid tribe Bathybatini by comparing phylogenetic trees of *Hemibates* and *Bathybates* species obtained with nuclear multilocus AFLP data with a phylogeny based on mitochondrial sequences. Consistent with our hypothesis, largely congruent tree topologies and negative tests for introgression provided no evidence for introgressive hybridization between the deepwater taxa. Together, the nuclear and mitochondrial data established a well-supported phylogeny and suggested ecological segregation during speciation.

## 1. Introduction

Cichlid fishes have undergone spectacular radiations in different parts of the world. In particular, the species flocks of the East African Great Lakes are well-known examples for rapid evolution and speciation [1–5]. Each of the three Great Lakes—Tanganyika, Malawi, and Victoria—is inhabited by hundreds of mostly endemic cichlid species [6, 7]. Notably, most of the diversity is found in the littoral habitat, whereas reduced species richness in the deep benthal and pelagial seems to be a common phenomenon in all East African Great Lakes [7–10]. At least three factors may have contributed to this pattern: (i) reduced niche diversity in the pelagic and in deepwater benthic zones, (ii) a narrow ambient light spectrum consisting only of short-wavelength blue light and hence less promotive of diversification mechanisms contingent on color perception than the shallow clear-water habitats [11–14], and (iii) the absence of strong barriers to gene flow. Indeed, deepwater cichlid species often have lake-wide distributions with very low, if any, population genetic structure over large geographic distances [10, 15, 16] (see also the Lake Tanganyika clupeid *Limnothrissa miodon* [17] and the centropomid *Lates stappersii* [18]). On the other hand, high levels of genetic differentiation, sometimes accompanied by phenotypic divergence on small geographic scales, are characteristic for the species-rich guild of stenotopic rock-dwelling cichlid species [19–31]. However, allopatric diversification in the fragmented littoral zone was not necessarily accompanied by the evolution of pre- or postzygotic isolation, so that secondary contact imposed by lake level fluctuations has often led to hybridization or introgression between previously allopatric taxa [32–35]. Even today, substrate breeders of the tribe Lamprologini can be found in mixed-species pairs [36], and interspecific fertilizations occur in communally nesting, shell-breeding lamprologines [37]. Indeed, phylogenetic analyses of predominantly littoral cichlid lineages revealed that interspecific hybridization has played, and still plays, an important role in the evolution of these fish [32, 34, 36–41]. Thus, in the majority of recent molecular studies on species relationships within littoral tribes, especially when comparing mitochondrial and nuclear phylogenies, the explanation for the tree topologies involved the claim of introgression and hybridization between established lineages in addition to incomplete lineage sorting (reviewed in [42]).

We hypothesize that this is not the case in tribes composed of deepwater species. Lacking the geographic structure introduced by littoral habitat heterogeneity, deepwater species may still be spatially separated by distance, by segregation of breeding grounds, by variable hydrological conditions [16, 43, 44], or by large-scale fragmentation of the lake basin during major droughts [45, 46]. Generally, however, the potential barriers to gene flow for deepwater species are less insurmountable than those met by stenotopic littoral cichlids. Specifically in Lake Tanganyika, the evolution of stenotopy regarding depth, bottom type, or light intensity may have been prohibited by the seasonal upwelling of anoxic waters [47]. We postulate that given the high potential for gene flow, diversification of lineages will either be curtailed (as suggested by the relative species paucity) or be attended by strong reproductive isolation right from the start. This would imply that introgression following cladogenesis occurred at much lower rates, if at all, in the deepwater species than in littoral cichlids. We test this hypothesis in the deepwater cichlid tribe Bathybatini by comparing phylogenetic trees based on mitochondrial sequence data with trees obtained with nuclear multilocus AFLP data, an approach which has previously revealed hybridization in littoral cichlids and other contexts [34, 36, 37, 39, 40, 48–54]. Moreover, in several studies, phylogenetic inference on species relationships has benefited from the use of multilocus genetic data, and we expect that the AFLP data collected in the present study will also contribute to the resolution of intergeneric relationships within the Bathybatini, which are still debated due to conflicting or ambiguous molecular and morphological evidence [46, 55–59].

### 1.1. The Study Species

The tribe Bathybatini (sensu Takahashi [59]) comprises 17 species in three genera: (1) the genus *Bathybates* comprises six large (30–40 cm), piscivorous species preying mainly on pelagic freshwater clupeids (*B. fasciatus* and *B. leo*), benthic cichlids (*B. graueri*, *B. vittatus*, and *B. ferox*), or undefined prey (the rare, elusive *B. horni*), in addition to the small (20 cm) *B. minor*, which is a specialized clupeid hunter. In accordance with their trophic niches, Coulter [43] distinguished three morphotypes among the *Bathybates* species, the fast-swimming fusiform predators *B. fasciatus*, *B. leo*, and *B. horni*, the generalized shape of the benthic feeders *B. graueri*, *B. vittatus*, and *B. ferox*, and the small clupeid-mimicking *B. minor*, which mingles with its prey and accompanies the diurnal clupeid migrations. Based on trawl net and gill net catches, *B. minor* were classified as pelagic, *B. fasciatus* and *B. leo* as chiefly bathypelagic and the remaining four species (*B. graueri*, *B. vittatus*, *B. ferox*, and *B. horni*) as chiefly benthic [43]. Except for *B. minor*, which was never found below 70 m, *Bathybates* species descend to depths of 150–200 m. (2) The member of the monotypic genus *Hemibates*, *H. stenosoma*, is an abundant benthic species on the muddy bottom of southern Lake Tanganyika feeding on fish and shrimps mainly at depths between 100 and 200 m [43]. (3) The small-bodied (<15 cm) species of the genus *Trematocara* (formerly assigned to the genera *Trematocara* and *Telotrematocara*, [55]) comprise nine benthic and bathypelagic species feeding on a variety on invertebrate prey, fish larvae, and phytoplankton. They are found at maximum depths of 75 to 200 m [43]. Following the upward movement of zooplankton, many *Trematocara* species undertake nightly migrations along slopes into the littoral.

All members of the Bathybatini are maternal mouthbrooders. Some species release their fry in shallow areas, but overall, data on bathybatine breeding behaviour is anecdotal or lacking [43, 60]. The species are sexually dimorphic, with males of *Bathybates* and *Hemibates* exhibiting species-specific patterns of dark stripes, bars and dots on a silver background and egg-spots on the anal fins, and males of the silvery *Trematocara* with dark dorsal fin markings. Females of all species show a uniformly silver/brown coloration. All Bathybatini have large eyes, which promote not only the detection of prey and predators but possibly also mate-recognition in the dark depths. In line with the latter, the monochromatic patterning of males may be viewed as adaptation to the short-wavelength dominated visual environment [61], in contrast to the colourful patterns of the mouthbrooding cichlids in the shallow, light-flooded littoral.

**Figure 1:**
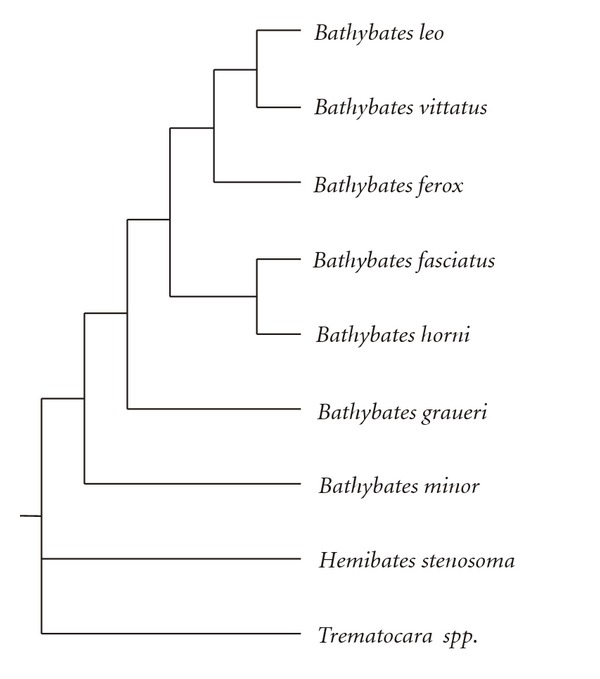
Schematic depiction of the the phylogenetic relationships within the Bathybatini as inferred from mtDNA data [46].

A recent phylogenetic study based on three mitochondrial genes supported the monophyly of *Bathybates* as well as of the species therein and indicated a polytomy of three equidistant lineages representing *Bathybates*, *Hemibates*, and *Trematocara* [46]. Within *Bathybates*, *B. minor* appeared ancestral to a radiation of the six large species, which showed a basal split of *B. graueri *and low statistical support for the branching order of the remaining species (Figure 1). The short internal branches among the large *Bathybates* species supported a rapid radiation at approximately 2.3–2.7 MYA, coinciding with the rapid diversification of other Lake Tanganyika cichlids [28, 45, 62]. Competition and resource partitioning as well as potential geographic isolation during an extreme low-stand of the lake were proposed as promoters of *Bathybates* speciation [46].

## 2. Material and Methods

### 2.1. Sample Collection and DNA Extraction

 This study is based on a total of 38 specimens, representing all seven *Bathybates* species, *Hemibates stenosoma* as well as *Trematocara unimaculata* and* T. macrostoma* (Table 1). All specimens were obtained between 1999 and 2011 from local fishermen at Lake Tanganyika and identified to species by S. Koblmüller. Unfortunately, it was not possible to obtain a comprehensive taxon sampling for the genus *Trematocara* as, because of their small size and hence low market value, these fish (except for the largest species *T. unimaculatum*) are not caught by local fishermen.

Fin clips or white muscle tissue were preserved in ethanol and DNA was isolated using a proteinase K digestion/high salt precipitation method [63]. DNA concentrations were measured using a NanoPhotometer (IMPLEN).

### 2.2. AFLP Data Collection

Amplified fragment length polymorphism (AFLP) genotyping followed the protocol described in [34]. The ten primer combinations used for selective amplification were *EcoR*I-ACA/*Mse*I-CAA, *EcoR*I-ACA/*Mse*I-CAG, *EcoR*I-ACA/*Mse*I-CAC, *EcoR*I-ACA/*Mse*I-CAT, *EcoR*I-ACT/*Mse*I-CAT, *EcoR*I-ACT/*Mse*I-CAA, *EcoR*I-ACT/*Mse*I-CAG, *EcoR*I-ACT/*Mse*I-CAC, *Eco*RI-ACC/*Mse*I-CAA, and *EcoR*I-ACC/*Mse*I-CAC. Selective amplification products were visualized using an ABI 3130xl automated sequencer (Applied Biosystems) along with an internal size standard (Genescan-500 ROX; Applied Biosystems). Polymorphic positions were initially identified using GeneMapper 3.7 software (Applied Biosystems) in a range of 50–500 bp. In order to adjust misaligned bins and avoid size homoplasy, bin positions were set manually. Bins containing ambiguous low intensity peaks in a large proportion of the samples and entire profiles with short read-lengths or very low peak heights were deleted. These preprocessed, un-normalized peak-height data were analyzed with AFLPScore 1.4a [64], which optimizes thresholds for locus retention and phenotype calling based on estimated error rates. Phenotype calling thresholds were set as absolute or relative depending on the number of retained loci and the achieved error rates obtained with each option. 20 replicate samples were included to calculate the mismatch error rate for all unique loci.

### 2.3. Phylogenetic Inference

A neighbour joining (NJ) tree based on Nei and Li's distances [65] was constructed in PAUP 4.0b5 [66]. Bootstrap values from 1000 pseudoreplicates were used as standard measure of confidence in the inferred tree topology.

Although accurate models for Bayesian tree construction using AFLP datasets do exist [67]), the high demands for processing power make them unfeasible to use [67, 68]. Thus, Bayesian phylogenetic inference (BI) was conducted in MrBayes 3.1.2 [69], employing the restriction site model with the “noabsencesites” coding bias correction [68, 70]. The Dirichlet prior for the state frequencies was set to (2.44, 1.00) matching the actual 0/1 frequencies in the dataset. Posterior probabilities were obtained from Metropolis-coupled Markov chain Monte Carlo simulations (2 independent runs; 10 chains with 8,000,000 generations each; chain temperature: 0.2; sample frequency: 1,000; burn-in: 4,000,000 generations). Chain stationarity and run parameter convergence were checked in Tracer 1.5 [71].

**Figure 2:**
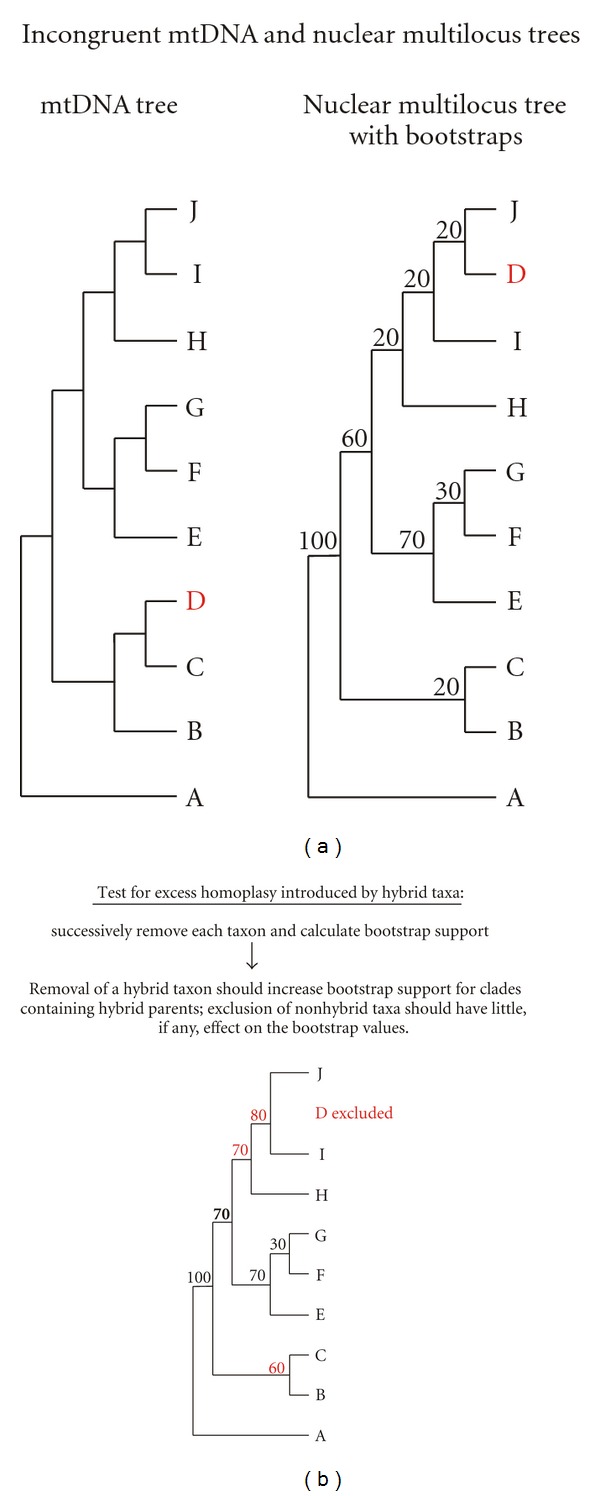
Incongruency between mtDNA and nuclear multilocus trees (e.g., AFLPs) and a test for hybridization in a multilocus phylogeny. (a) Incongruency between mtDNA and nuclear multilocus trees with regard to the placement of taxon D can result from ancient incomplete lineage sorting [72, 73] or the hybrid origin of taxon D. (b) As hybrid taxa combine nuclear alleles from both parental taxa, they introduce homoplasy into a multilocus phylogenetic tree and hence reduce bootstrap support of the nodes containing their parents [74]. Removal of the hybrid taxon from the phylogeny increases the bootstrap support of the parental clades (bold values in (b)). Conversely, removal of nonhybrid taxa should not or only slightly affect the bootstrap support of other nodes. To distinguish between informative (red values in (b)) and uninformative changes in bootstrap values, one taxon at a time is removed from the data and the resulting distribution of bootstrap values for each node is recorded. If removal of a certain taxon produces an outlier in these distributions, the removed taxon is considered a hybrid (or strongly introgressed taxon) and the clades for which support was raised are considered to contain the parental taxa (see, e.g., [34, 40, 48]). In the present example, taxon D is a hybrid between taxa C and J.

To test for homoplasy excess introduced by hybridization, we conducted a tree-based method as outlined by Seehausen [74] by removing single species from the dataset and observing the change in bootstrap values in the NJ tree (see also [34, 40]). In theory, the inclusion of a hybrid taxon in a multilocus phylogeny introduces homoplasy with clades that contain its parental taxa. Hybrid taxa should be intermediate to the parental taxa since they carry a mosaic of parental characteristics. Thus, decreasing the amount of homoplasy in the dataset by removing the hybrid taxon should increase the bootstrap support for the clades that include the parental taxa or their descendants, whereas removing nonhybrid taxa should have no effect on the statistical support of other nodes (Figure 2).

### 2.4. Evaluating Alternative Tree Topologies

Testing for consistency between mtDNA- and AFLP-based tree topologies employed two different strategies. In a first test we evaluated whether our AFLP-NJ-topology can be explained by the mtDNA data of Koblmüller et al. [46]. Using the mitochondrial sequences, we inferred the log likelihood of the AFLP-NJ-topology data by constraining maximum likelihood (ML) tree search to a topology identical to the species tree suggested by the NJ analyses of our AFLP data, applying the substitution model used in this previous study (HKY+I+G). To test for significant differences between the unconstrained [46] and constrained mtDNA topology we performed an ML-based Shimodaira-Hasegawa (SH) test [75] (full optimization, 1,000 bootstrap replicates) in PAUP. In a second test we evaluated by means of a Bayes factors approach [76] whether the mtDNA tree [46] can be explained by our AFLP data, and whether the AFLP-NJ and BI trees differ significantly from each other. We performed BI searches constraining the topology to that of the mtDNA-topology [46] and the interspecific relationships implied by the AFLP-NJ-tree in MrBayes 3.1.2 employing the same settings as above. Bayes factor comparison—using the harmonic means of the likelihood throughout different runs [77, 78]—among the three alternative phylogenatic hypotheses was performed in Tracer 1.5. Values of 2 × ln BF (two times the difference between the harmonic means of the two models) >10 are considered strong evidence for support of one model over another [76].

## 3. Results

**Figure 3:**
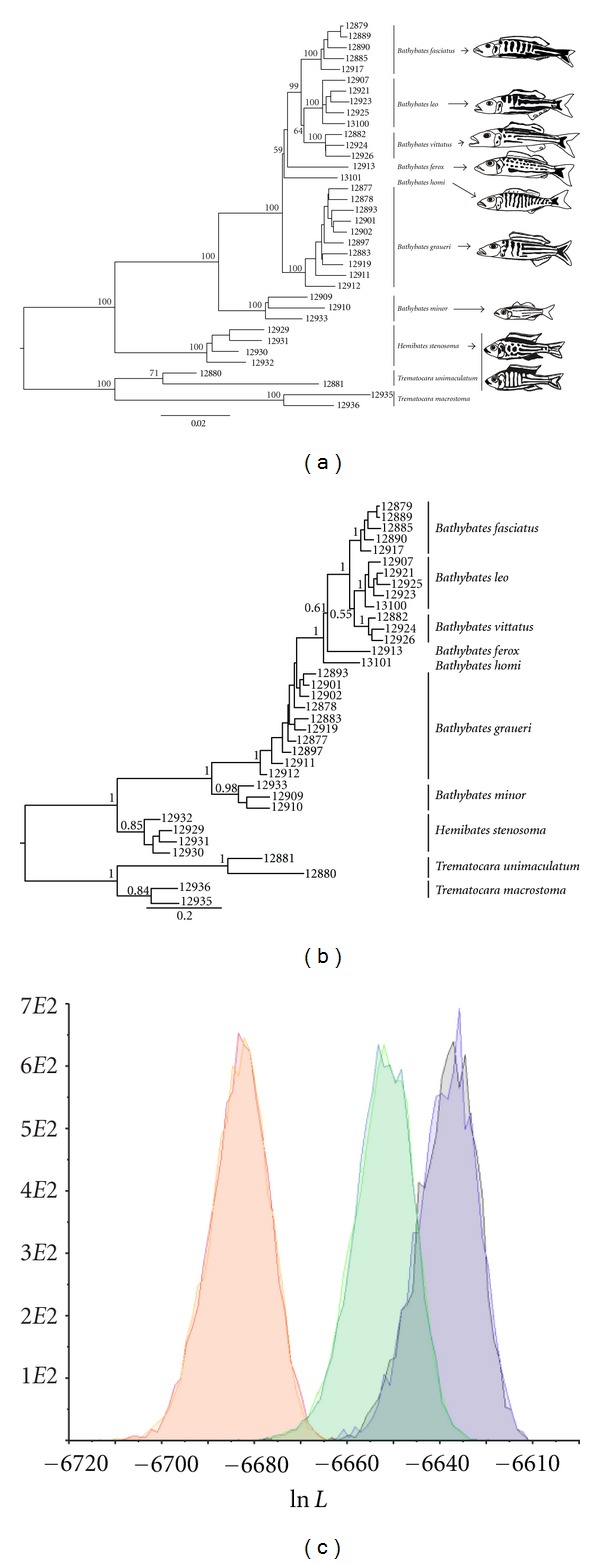
Phylogenetic relationships of the Bathybatini based on 659 polymorphic AFLP loci. (a) NJ tree (employing Nei and Li distances [65]) and (b) BI tree are shown. Only bootstrap values >50 and posterior probabilities >0.5 are shown. Branches are labelled with the samples' lab ID numbers. (c) The marginal density of posterior distribution of likelihood (Ln *L*) for each of the two MrBayes runs of the unconstrained AFLP data (violet, grey), the AFLP-NJ-topology-constraint (blue, green), and the mtDNA-topology-constraint (orange, red). Fish were drawn after photographs in [60] to demonstrate the interspecific differences in male nuptial patterns.

The final AFLP dataset consisted of 659 unique loci with a mismatch error rate of roughly 3%, which falls within the acceptable limit for mismatch error rates as defined by [64]. Both the NJ and BI analysis yielded largely congruent and well-supported topologies with only minor differences between them (Figures 3(a) and 3(b)). Whereas all species were monophyletic in the NJ tree, *Bathybates graueri* was not resolved as a monophylum in the BI tree, but as paraphylum including the well-supported clade of the other large *Bathybates* species (Figure 3(b)). Despite this minor topological difference, Bayes factor comparison strongly supports the BI tree over the NJ tree (2 × ln BF = 26.828; Figure 3(c)). We note, however, that the two-state model implemented in MrBayes does not fully cover the complex genetic process of AFLP evolution and thus provides accurate phylogenetic inference less likely than distance methods [67, 79, 80]. Hence, the observed differences between AFLP and NJ tree topologies might be attributed to this problem. Both the NJ and BI analyses support the monophyly of all three genera with the genus *Trematocara* representing the most ancestral branch (Figures 3(a) and 3(b)). Within the genus *Bathybates*, the small and morphologically most distinct member of the genus, *B. minor*, was sister taxon to the remaining large *Bathybates* species. Branch lengths among the large *Bathybates* species are rather short and some received rather low statistical support, indicating a period of rapid cladogenesis. Nevertheless, both NJ and BI analyses revealed a largely consistent phylogenetic pattern within the large *Bathybates* species. Both SH-test and Bayes factor comparison revealed significant differences between mtDNA and AFLP phylogenies (SH-test: ln *L* of −9505.385 versus −9585.142 for mtDNA versus AFLP-NJ-topology-constraint, *P* < 0.001; Bayes factors: 2 × ln BF of 88.576 between mtDNA and AFLP-BI-topology and 61.748 between mtDNA and AFLP-NJ-topology, Figure 3(c)). The homoplasy excess test provided no evidence for introgressive hybridization (data not shown).

## 4. Discussion

In the original classification of Lake Tanganyika cichlid tribes by Poll [55], *Bathybates* and *Hemibates* were included in a tribe Bathybatini as sister group to the Trematocarini (equivalent to the genus *Trematocara*), a hypothesis supported both by lepidological [58] as well as allozyme data [57]. In contrast, based on morphological characteristics, Stiassny [56] and Takahashi [59] proposed a sister group relationship of *Bathybates* and *Trematocara*. Currently, all three genera, *Bathybates*, *Hemibates*, and *Trematocara*, are united in the tribe Bathybatini [59]. A previous mtDNA phylogenetic study remained equivocal with regard to the two competing morphological classifications and suggested that *Bathybates*, *Hemibates,* and *Trematocara* diverged rapidly from their common ancestor [46]. In the present AFLP phylogeny, the length differences between the most ancestral branches favour Poll's original classification with *Trematocara* as sister group to *Hemibates* + *Bathybates* [55, 57, 58]. Consistent with the mitochondrial phylogeny [46], the AFLP data confirm the split between *Bathybates minor* and the larger members of the genus *Bathybates* with *B. graueri* as their most basal representative and identifies a period of rapid cladogenesis at the onset of the diversification of the large *Bathybates* species. However, mtDNA and AFLP phylogenies differ significantly with respect to the branching pattern among the remaining large *Bathybates* species. Introgressive hybridization (including the possibility of complete mtDNA replacement [41]) and ancient incomplete lineage sorting are two alternative sources of topological disagreement between nuclear and mitochondrial trees [40, 41, 81–83], resulting in similar phylogenetic patterns that are difficult to resolve by strict hypothesis testing [84]. Circumstantial inference can be based on the fact that lineage sorting is expected to lag behind rapid cladogenetic events, such that the rapid radiation of the large *Bathybates* species predisposes this clade to mitonuclear phylogenetic incompatibilities without implying postcladogenetic introgression [72, 73]. Likewise, monophyly of species in both mitochondrial and nuclear trees (excepting the paraphyly of *B. graueri* in Bayesian AFLP tree) and negative tests for homoplasy excess in the AFLP data do not indicate the presence of hybrid taxa in the genus *Bathybates*. These findings support our hypothesis that deepwater species are less prone to introgressive hybridization and hybrid speciation than littoral species, but reservations arise on the one hand from the possibility that events of introgression were not detected in our samples and data and given the power of our analyses, and on the other hand from the small number of species in the phylogeny. Principally, rates of interspecific introgression may not differ between littoral and deepwater cichlids, but will nonetheless lead to higher incidences of introgression in the species-rich groups than in a less speciose clade. If this was the case, the lack of a signal of introgression in *Bathybates* and *Hemibates* would be fully explained by the low diversification rate of the lineage and hence limited opportunity for interspecific hybridization, without implying the evolution of complete reproductive isolation early on during diversification.

The branching order of *Hemibates* and the basal *Bathybates* species, which is reconstructed congruently by mtDNA and AFLP markers, suggests repeated transitions between benthic and bathypelagic feeding mode (ecological data from [43]). The basal *H. stenosoma* represents a benthic generalist feeding on shrimps and various species of fish. The next split led to the specialized pelagic clupeid hunter *B. minor*, which mimics its prey in size and coloration and stages surprise attacks from within the sardine shoals. Then, *B. graueri* took a step back to the benthic habitat, specialized on cichlid prey and evolved a large body size. The chronology of the following radiation of the large bathypelagic clupeid hunters and benthic cichlid hunters remains unresolved, but involved at least one transition from benthic to bathypelagic habitat preferences. Depth preferences may vary between these species [43], such that speciation may have involved both niche and spatial segregation. The apparent ecological differentiation among lineages may have reduced the fitness of hybrids [85–87] and may have promoted the evolution of a mate recognition system, perhaps based on the species-specific melanic patterns of male *Hemibates* and *Bathybates*. The efficacy of monochromatic black, silvery, and white body and fin patterns in mediating assortative mating in the dark, short-wavelength dominated environment has recently been demonstrated for deepwater cichlid species of Lake Malawi. These sympatric and morphologically similar species differ primarily in male nuptial patterns and their reproductive isolation is corroborated by genetic differentiation estimates [61]. However, there is increasing evidence that color pattern is not the only cue for mate recognition in cichlids fish and it is possible and likely that auditory [88] and olfactory cues [89] play a role in mediating assortative mating in deepwater species, too.

## 5. Summary and Conclusions

In concert with previous mitochondrial data, the present study provides an informative phylogeny of the species in the deepwater genera *Hemibates* and *Bathybates*. As far as the branching pattern can be resolved, it suggests ecological segregation during speciation. The rapid radiation within *Bathybates* mirrors a burst of speciation observed in several other cichlid tribes of Lake Tanganyika and reveals a congruent cladogenetic pattern across vastly different habitats, which suggests some kind of synchronization by environmental factors [27, 45, 46, 62]. Consistent with the hypothesis that lineages evolving in the weakly structured deepwater habitat would develop stronger reproductive isolation than the allopatric lineages of the fragmented littoral, our data provided no evidence for the presence of hybrid taxa in the deepwater dwelling genus *Bathybates.* In further support of the hypothesis, introgressive hybridization is also not indicated by the mitochondrial and AFLP phylogenies of the Lake Tanganyika cichlid genus *Xenotilapia* [90], which includes several deepwater-dwelling species comprising the prey of the benthic *Bathybates* and *Hemibates*. An increased sample size to evaluate this pattern will be attained by the analyses of additional open-water and deepwater species, for example, the tribe Limnochromini and the genus *Trematocara*.

## Figures and Tables

**Table 1:** List of samples with sample ID and sampling locality.

Sample ID	Species	Sampling locality
12879	*Bathybates fasciatus*	Mpulungu market
12885	*Bathybates fasciatus*	Mpulungu market
12889	*Bathybates fasciatus*	Kalambo Lodge
12890	*Bathybates fasciatus*	Kalambo Lodge
12917	*Bathybates fasciatus*	Tanganyika Lodge
12913	*Bathybates ferox*	Lufubu estuary
12877	*Bathybates graueri*	Mpulungu market
12878	*Bathybates graueri*	Mpulungu market
12883	*Bathybates graueri*	Mpulungu market
12893	*Bathybates graueri*	Mpulungu market
12897	*Bathybates graueri*	Mpulungu market
12901	*Bathybates graueri*	Mpulungu market
12902	*Bathybates graueri*	Mpulungu market
12911	*Bathybates graueri*	Mpulungu market
12912	*Bathybates graueri*	Mpulungu market
12919	*Bathybates graueri*	North of Sumbu
13101	*Bathybates horni*	Mpulungu market
12907	*Bathybates leo*	Mpulungu market
12921	*Bathybates leo*	Mpulungu market
12923	*Bathybates leo*	Mpulungu market
12925	*Bathybates leo*	Mpulungu market
13100	*Bathybates leo*	Mpulungu market
12909	*Bathybates minor*	Lufubu estuary
12910	*Bathybates minor*	Kalambo
12933	*Bathybates minor*	Sumbu
12882	*Bathybates vittatus*	Mpulungu market
12924	*Bathybates vittatus*	Mpulungu market
12926	*Bathybates vittatus*	Mpulungu market
12929	*Hemibates stenosoma*	Mpulungu market
12930	*Hemibates stenosoma*	Mpulungu market
12931	*Hemibates stenosoma*	Mpulungu market
12932	*Hemibates stenosoma*	Mpulungu market
12880	*Trematocara unimaculata*	Mpulungu market
12881	*Trematocara unimaculata*	Mpulungu market
12935	*Trematocara macrostoma*	Mpulungu market
12936	*Trematocara macrostoma*	Mpulungu market

Coordinates of sampling sites (if known): Kalambo, S 8°37′ E 31°12′; Kalambo Lodge, S 8°37′ E 31°37′; Lufubu estuary, S 8°32′ E 30°44′; Sumbu, S 8°31′ E 30°29′; Tanganyika Lodge, S 8°47′ E 31°05′.

Note that fish obtained at the fishmarket in Mpulungu might have been caught anywhere in southern Lake Tanganyika.
